# Highly Sensitive Hot-Wire Anemometry Based on Macro-Sized Double-Walled Carbon Nanotube Strands

**DOI:** 10.3390/s17081756

**Published:** 2017-08-01

**Authors:** Dingqu Wang, Wei Xiong, Zhaoying Zhou, Rong Zhu, Xing Yang, Weihua Li, Yueyuan Jiang, Yajun Zhang

**Affiliations:** 1 Institute of Nuclear and New Energy Technology, Tsinghua University, Beijing 100084, China; xwthu@tsinghua.edu.cn (W.X.); liweihua@tsinghua.edu.cn (W.L.); jiangyy@tsinghua.edu.cn (Y.J.); yajun61@tsinghua.edu.cn (Y.Z.); 2 Collaborative Innovation Center of Advanced Nuclear Energy Technology, Tsinghua University, Beijing 100084, China; 3 Key Laboratory of Advanced Reactor Engineering and Safety, Ministry of Education of China, Tsinghua University, Beijing 100084, China; 4 State Key Laboratory of Precision Measurement Technology and Instrument, Department of Precision Instrument, Tsinghua University, Beijing 100084, China; zhouzz@tsinghua.edu.cn (Z.Z.); yangxing@tsinghua.edu.cn (X.Y.)

**Keywords:** hot-wire anemometer, flow-rate sensor, carbon nanotube, highly sensitive

## Abstract

This paper presents a highly sensitive flow-rate sensor with carbon nanotubes (CNTs) as sensing elements. The sensor uses micro-size centimeters long double-walled CNT (DWCNT) strands as hot-wires to sense fluid velocity. In the theoretical analysis, the sensitivity of the sensor is demonstrated to be positively related to the ratio of its surface. We assemble the flow sensor by suspending the DWCNT strand directly on two tungsten prongs and dripping a small amount of silver glue onto each contact between the DWCNT and the prongs. The DWCNT exhibits a positive TCR of 1980 ppm/K. The self-heating effect on the DWCNT was observed while constant current was applied between the two prongs. This sensor can evidently respond to flow rate, and requires only several milliwatts to operate. We have, thus far, demonstrated that the CNT-based flow sensor has better sensitivity than the Pt-coated DWCNT sensor.

## 1. Introduction

To date, hot-wire (or hot film) anemometers (HWA) [1,2], which have been used for more than 100 years, have typically been made of platinum (Pt) or tungsten wires, and are usually hundreds of microns long and about 5 μm in diameter, which dictates its spatial resolution. In the field of fluid measurement, HWAs are predominantly used for measurement of turbulence and unsteady laminar flow. It is well known that the dimensions or the surface-to-volume (S/V) ratio of the material structure greatly influence its sensing performance [3], especially for thermal sensing, because of the difference in the heat exchange area [4]. 

Carbon nanotubes (CNTs), which possess remarkable mechanical, thermal and electrical properties [5,6,7,8], have been widely utilized for sensing in applications such as motion sensors [9], temperature sensors [10,11] and flow measurement [12,13,14,15,16,17]. Generally, the potential applications for CNTs are ascribed to their fancy physical and chemical properties. Nevertheless, most efforts have been focused on exploring the applications of an individual CNT; bulk or bundled CNTs have rarely been involved in the research [14,18]. Recently, some attention has been given to macroscopic assemblies of carbon nanotubes (CNTs), including bucky-papers, fibers, pellets and thin films, in order to utilize the characteristic properties of a single CNT on a macroscopic scale [12,16]. Because of the larger size of macro CNTs, it is possible to maintain them in batches without micro-operation. In recent years, the thermal correlation sensing properties of CNTs have been widely investigated, owing to their large S/V, low power consumption and high sensitivity. Consequently, utilizing macro-size CNTs as hot wires is a natural idea, and has been developed for sensing flow-rate in recent years. It was believed that the high aspect ratios and large surface area of nanotubes could be beneficial to the thermal conduction in the flow medium (e.g., air). 

In our earlier study [12] of hot-wire flow sensors with multi-walled carbon nanotube strand sensing elements, the calculated sensitivity of the sensor was 3.38% under flow velocity of 0 to 15 m/s, which is about 80 mV·s/m at low velocity. Hsu M. et al. [15] reported a type of hot-film anemometer, with a sensitivity calculated from the slope of the voltage difference against the windspeeds curve that was about 0.4099 mV·s/m. Ito Y. et al. [4] developed a sub-microscale flow sensor that consisted of a suspended Pt hot-film and CNT fins, and which utilized the large S/V of CNT to enhance the heat transfer and improve the flow measurement sensitivity to about 20 mV·s/m. Dinh T. et al. [17] reported on a low-cost, environmentally-friendly and wearable thermal flow sensor, which offered excellent performance, such as high signal-to-noise ratio (≥40 dB for an air flow velocity of 1 m/s) and high sensitivity to airflow (53.7 mV(m/s)^−0.8^). All of these works show the device’s potential for nanotubes as sensitive flow sensors. 

In this paper, we study a flow measurement with much higher sensitivity than the aforementioned ones, consisting of a thermal self-heating sensor of micro-sized CNT strands, which consist of tens of thousands of parallel DWNT [19]. Furthermore, much higher sensitivity than conventional Pt HWA was observed in the experiments. 

## 2. Theoretical Analysis

HWA is a typical electromechanical system that is based on the change of the electrical resistance of the hot wire with the changing wire temperature. Fluid rate is measured by sensing changes in heat transfer from an electrically heated hot wire exposed to the fluid. The HWA can be operated in different modes, the two most important of which are constant current (CC) and constant temperature (CT) [1,2]. Using constant current dissipation means that the temperature of the heated hot wire decreases with increased flow rate. 

In steady state, the output characteristics of an anemometer should follow King’s law [2],

(1) $$IV=\left(T-T_{a}\right)\left(K_{0}+K_{1}U^{n}\right)$$

where *I* and *V* are the current and voltage drop across the wire, *T* is the average wire temperature, *T*_a_ is the ambient temperature, *U* is the flow velocity, n depends on the wire geometry and is about 0.5 for conventional hot wires, *K*_0_ and *K*_1_ are the constants related to convective heat transfer and can be experientially expressed as ref. [2],

(2) $$K_{0}=0.42\pi kl{\left(\frac{\mu C_{p}}{k}\right)}^{0.2}$$

(3) $$K_{1}=0.57\pi kl{\left(\frac{\mu C_{p}}{k}\right)}^{0.33}{\left(\frac{\rho d}{\mu }\right)}^{n}$$

where *k* is the thermal conductivity of the gas, *μ* is dynamic viscosity of the gas, *C*_p_ is the specific heat of the gas at constant pressure, *ρ* is the gas density, and *d* is the wire diameter. 

When the HWA work in CC mode, it can then be derived from Equation (1),

(4) $$V_{0}-V=\frac{AU^{n}}{1+BU^{n}}$$

where *A* and *B* are velocity-independent constants, and can be gained by fitting the experimental data. Another form of the expression is as follows,

(5) $$V_{0}-V=\frac{\alpha IR_{a}\left(T-T_{a}\right)}{K_{0}-\alpha I^{2}R_{a}}K_{1}U^{n}$$

where *α* is the TCR of the hot wire, *I* is the constant current, *T* is the operate temperature, *R*_a_ is the wire resistance at ambient temperature, and *V*_0_ is the voltage drop across the wire at zero fluid. Equation (5) reveals that larger *K*_1_, *T* and *α* is the key for attaining higher flow sensitivity of anemometer.

## 3. Experimental Details

**Figure 1:**
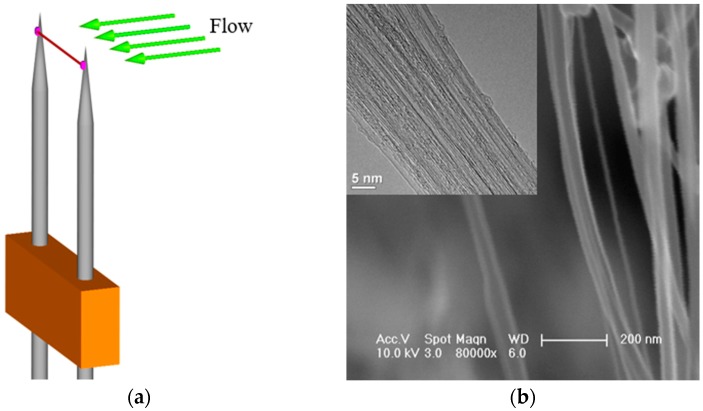
(**a**) Schematic illustration of an HWA; (**b**) SEM image of a DWCNT strand. The inset is a HRTEM image.

The centimeters long strands of double-walled carbon nanotubes (DWCNT) used in our experiments, which were prepared by Jinquan Wei [19], were synthesized by catalytic chemical vapor deposition method in a quartz tube. Figure 1b shows typical SEM and TEM images of the DWCNT. The diameter of the DWCNT bundles is primarily distributed in a range of 5–30 nm. Few impurities are observed in the image, which indicates the high purity of the DWCNT in the samples.

### 3.1. Assembly Process

Firstly, the DWCNT strands were pulled out along their length using precise tweezers to obtain thinner strands with diameters of about 20 μm. Then, the DWCNT strands were suspended directly on two tungsten prongs. In order to fix the two ends of the strand and improving the contact characteristics between the strand and the prongs, a small drop of silver glue was dripped on each contact area. The silver glue was dried in the shade for about 24 h, after which the anemometer was completely constructed to survey the air speed. Figure 1a is a representative schematic illustration of the anemometer with a DWCNT hot-wire.

### 3.2. Hot-Wire Anemometry Test Facility

The steady-state performance of the anemometry sensor was tested in a wind tunnel. The maximum air velocity in the wind tunnel was about 15 m/s, which was calibrated by a commercial HWA. At this stage, we studied the responses of the anemometers under different air velocities operated in CC mode mainly from 0 to 10 m/s. A SMU 237 (Source Measurement Unit 237, Keithley, Beaverton, OR, USA) was employed to apply the constant current to the anemometer and measure its output voltage. 

## 4. Results and Discussion

### 4.1. Overheating Characteristics

**Figure 2:**
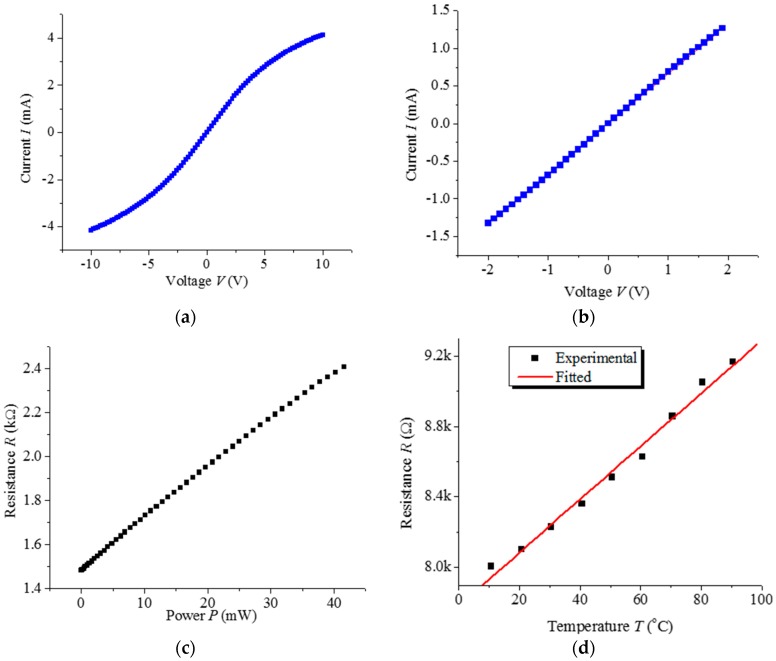
(**a**) The flexual I-V characteristic and the straight line are the experimental measurement of a DWCNT HWA and the theoretical expectation using Ohm’s Law under a bias from −10 V to 10 V; (**b**) The I-V characteristic of the same specimen with a bias from −2 V to 2 V. The room temperature resistance of the sensor of this specimen was about 1.5 kΩ; (**c**) Deduced resistance of DWCNT strand specimen related to applied electrical power; (**d**) Experimental and fitted resistances of another DWCNT strand specimen related to temperature.

We determined the I-V characteristics of the DWCNT HWA for investigating the self-heating phenomenon by using the SMU 237. As shown in Figure 2, the anemometer was characterized by applying a bias from −10 V to 10 V, and from −2 V to 2 V. Figure 2a demonstrates that the I-V characteristic decreases with an increase in applied voltage. The reason for this phenomenon is the self-heating of the DWCNT under a higher voltage. The deflexed experimental curve implies that the resistance of DWCNT rises with increasing temperature. This consequently indicates that the temperature coefficient of resistance (TCR) of the DWCNT is positive, which is significantly different from the negative TCR of reported single-walled CNTs and multi-walled CNTs [20,21,22]. When the applied voltage is limited to a small range, the I-V characteristic nearly represents a line. According to the linear characteristic, the contact barriers between the DWCNT and the electrode are very low and can be neglected. On the basis of the I-V characteristic in Figure 2a, the deduced resistances under various degrees of applied electrical power are shown in Figure 2c. The near-linear relationship between the temperature of the heating wire and the applied power accords with the law of conservation of energy.

The thermal characteristics of the device are tested under various temperature conditions in a controlled oven. The oven temperature is set by the built-in computer, ranging from 10 to 90 °C. Because of the self-heating effect under a large bias, a small voltage is applied for measuring the resistance at a specific temperature, when measuring its TCR. The I-V measurement at low bias voltages ranging from −0.5~0.5 V for different temperatures is carried out to deduce the resistance of the DWCNT. The deduced DWCNT resistances for different temperatures are shown in Figure 2d.

**Figure 3:**
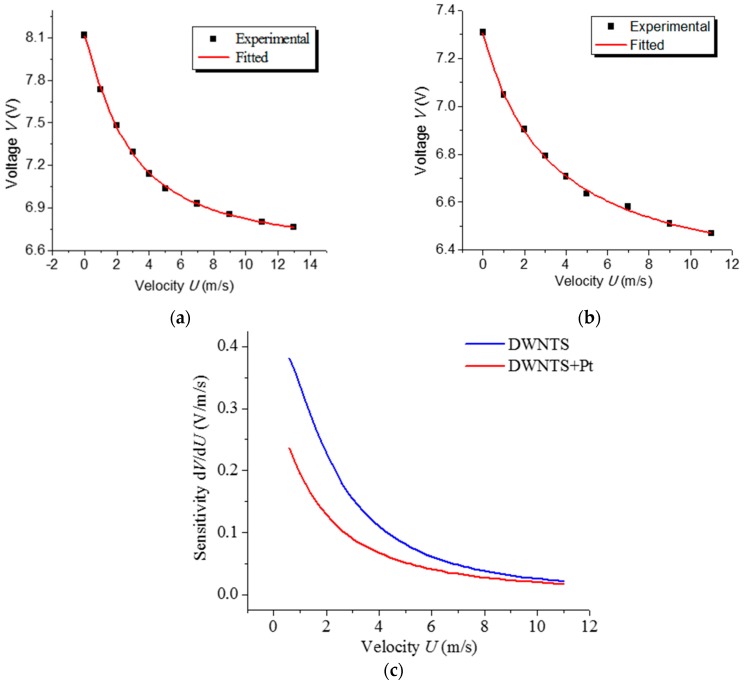
Output voltages and fittings by Formula (4) of an anemometer under diversified air velocity. (**a**) DWCNT hot wire, fitted exponent *n* = 1.21; (**b**) DCWNTs hot wire deposited by 10 nm Pt, fitted exponent *n* = 1.01; (**c**) Sensitivity of DCWNTs and Pt-coated DWCNT hot-wire in CC mode.

The relationship between temperature and resistance for the DWCNT is generally described as

(6) $$R=R_{a}\left[1+\alpha \left(T-T_{a}\right)\right]$$

where *R* is the resistance at temperature *T*, *R*_a_ is the resistance at ambient temperature *T*_a_ (e.g., 20 °C), and *α* is the TCR. As shown in Figure 3, by fitting the experimental data using (6), the TCR of the DWCNT is derived to be about 1980 ppm/K, which is positive and much larger than that of the multi-walled carbon nanotubes reported in [23]. 

This result reveals that DWCNT have good potential for use in thermal sensing. From the experimental results, the DWCNT behave as a positive TCR, which is in agreement with the above analysis.

As shown in Figure 2a, the self-heating effect is more and more evident with an increase in applied voltage, because of the quadratically afferent power. Consequently, an anemometer relying on the self-heating effect should be operated under a not-too-small constant current to heat the wire to a higher temperature. 

### 4.2. Performance of Hot-Wire Flow Sensors

Figure 3a shows the steady state characteristics of the DWCNT HWA. Due to the positive TCR, the voltage across the hot wire decreases as the flow rate increases. Curve fitting with (4) verifies good correspondence between the measured data and the theoretical relation, where *n* equals 1.21. However, commercial HWAs have a geometric exponential factor of about 0.5, which means more degradation at higher velocity, compared to the DWCNT HWA presented here. In our estimation, this variation is probably due to its loose structure and large S/V ratio. Some previous studies [24] have obtained similar results with *n* up to 1.1.

For investigating the distinction of conventional HWA with a Pt hot-wire, Pt was deposited onto the DWCNT by PVD with a thickness of about 10 nm. Then, the Pt-coated DWCNT HWA was tested in the wind tunnel in CC mode. As shown in Figure 3b, the resistance of hot-wire is slightly lower, and the fitted curve dovetails nicely with the theoretical relation. The sensitivities of the DWCNT hot-wire and Pt-coated DWCNT hot-wire, defined as the derivative of output voltage, are also compared by their relation to air flow velocity (Figure 3c). Compared to the maximum sensitivity of 0.06 V/(m/s) of the commercial HWA shown in [2], we attain a maximum sensitivity of about 0.4 V/(m/s). The main reason for this advantage is that the CNT calculated operating temperature of up to more than 1000 °C, much higher than the ~300 °C of the commercial HWA. It’s remarkable that the sensitivity decreases after deposition of Pt on DWCNT. The difference in the sensitivity reveals the advantage of applying DWCNT as hot-wire over Pt. Once Pt was deposited on the DWCNT, the dominating hot-wire in the HWA was diverted to Pt because of its low resistivity.

**Figure 4:**
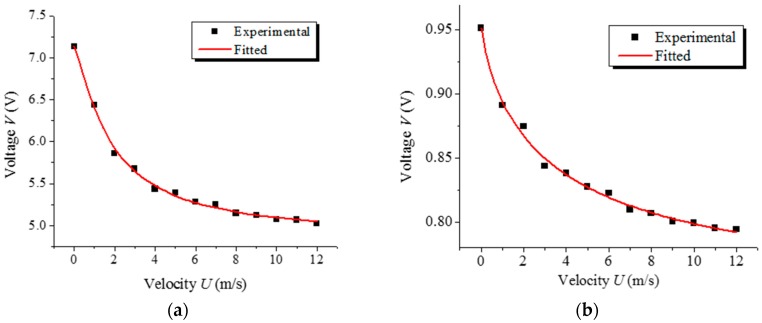

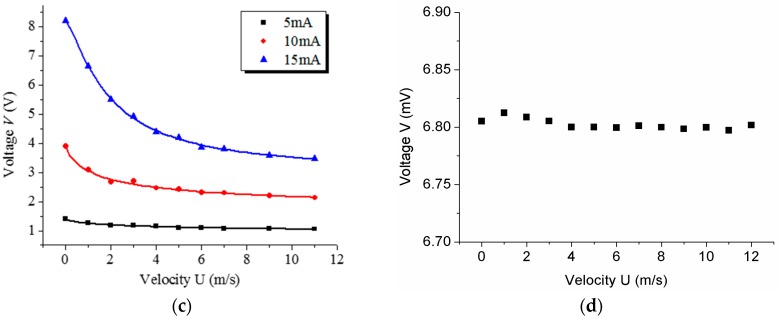
(**a**–**c**) Output voltages and fittings of two DWCNT anemometers under diversified air velocity with different driving currents; (**a**) sensor N1: 6 mA, fitted exponent *n* = 1.23; (**b**) sensor N1: 1 mA, fitted exponent *n* = 0.82; (**c**) sensor N2: 5 mA (*n* = 0.70), 10 mA (*n* = 0.77) and 15 mA (*n* = 1.29); (**d**) Output voltages of the flow sensor under diversified air velocity with a tiny driving current of 10 μA.

Subsequently, the influence of the working current is investigated in the experiments. The sensitivity of output voltage with respect to air velocity increases with increasing driving current because of the increase in the temperature difference between the hot wire and the fluid. In our experiments, different current is applied to the HWA to investigate its sensitivity. Figure 4a,b shows the anemometry of a DWCNT HWA with two different currents of 6 mA and 1 mA. Another three results are shown in Figure 4c, with different currents of 5 mA, 10 mA and 15 mA. All the results indicate that the voltage drop is much larger at a higher working current, but they all flatten out at high velocity. It can be seen that the experimental data tally closely with the theoretical relation in CC mode. In addition, the fitted exponents of expression (4) greatly increase with the applied power. 

When the sensor is tested under air flow, stress occurring at the CNT strands may lead to a resistance change as a result of the piezoresistance effect, since this effect is coupled with the thermal resistance change, which can significantly affect the performance of the sensor. For evaluating the influence, we applied a tiny driving current across CNT strands of 10 μA to avoid the overheat heat exchange. Then the voltage at the sensor is obtained under various air flows, as shown in Figure 4d. The almost horizontal curves indicate that there are no piezoresistance influences on the sensor.

**Figure 5:**
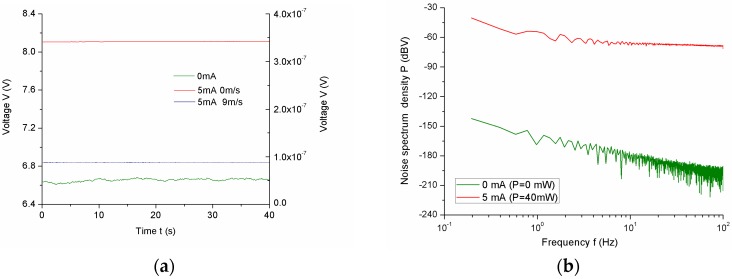

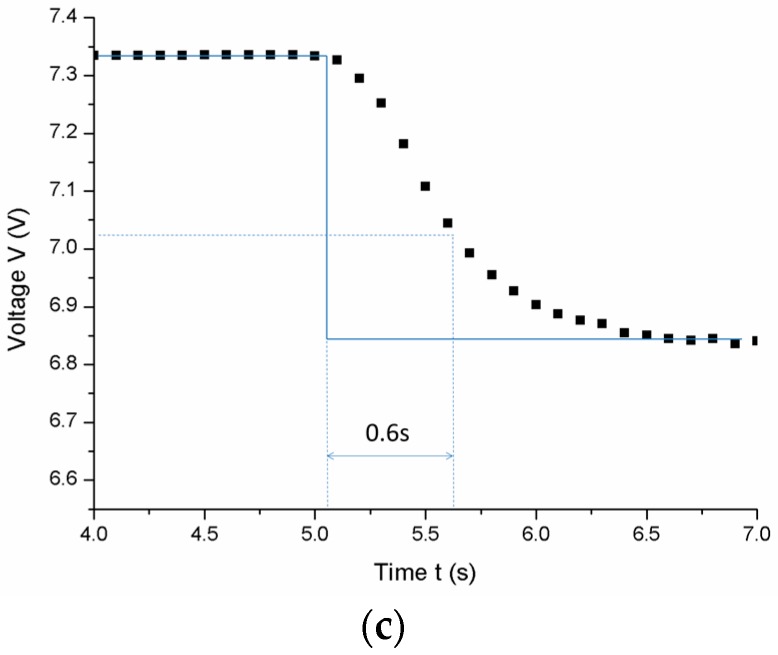
(**a**) Real-time response of the sensor under 0 mA and 5 mA; (**b**) Noise spectral density of the sensor; (**c**) Thermal response time of the sensor.

As a high signal-to-noise ratio (SNR) is desired for the flow sensor, we measured the noise of the sensor. The real voltage responses of the sensor under different applied currents are shown in Figure 5a. As shown in Figure 5b, the noise spectrum density increases with increased applied power, and decreases with increased working frequencies. Therefore, the SNR was calculated to be about 63, which is at least one order of magnitude higher than the standard SNR of a true signal (SNR = 3).

Due to the thermal inertia of the wire element, it will not respond instantaneously to changes in the flow condition when operated in CC mode. The response time can be estimated as for standard hot-wire [2],

(7) $$M=\frac{\rho_{W}c_{W}\left(\pi /4\right)d^{2}l\left(R_{W}-R_{a}\right)}{\alpha R_{0}I^{2}R_{a}}$$

where $\rho_{W}$ and $c_{W}$ are the density and specific heat, respectively, of the hot-wire, *R*_W_ is the resistance of heated wire. Because of the complicated internal structure and inaccurate performance parameters of the CNT strand, it’s hard to accurately calculate the response time. By coarsely searching for relevant parameters from the literature, the response time of DWCNT flow sensors is estimated to be about 0.6 ms. As it is impossible to stop the flowing fluid quickly enough by turning down the wind tunnel, the response time test of the sensor will be influenced by the fluid slowing down. As shown in Figure 5b, the thermal response time of the sensor is estimated to be 0.6 s, about 1000 times the theoretical value.

## 5. Conclusions

We developed a simple and highly sensitive air flow sensor with a double-walled CNT strand as the sensing element. The flow sensor was assembled by suspending the DWCNT strand directly on two tungsten prongs and a small volume of silver glue was dripped on each contact between the DWCNT and prongs. The TCR test showed that the DWCNT have a positive TCR of 1980 ppm/K. As a result of the self-heating effect of DWCNT, flow sensing by means of removing the heat in the strand and changing the temperature is feasible. The flow sensor was subsequently fabricated and tested. The results show that the sensor can respond to flow rate, and requires only several milliwatts to operate. We demonstrated that the CNT-based flow sensor has better accuracy than a Pt-coated DWCNT sensor.
